# Colorectal cancer disease profile and treatment patterns at an urban tertiary hospital in Rwanda

**DOI:** 10.3332/ecancer.2024.1687

**Published:** 2024-03-28

**Authors:** Margaret Baldwin, Brandon A Niyibizi, Daniella Rangira, Benita Rangira, Madeleine K Kazindu, Daniel Seifu, Cristina Daniela Stefan, Eulade Rugengamanzi, Achille V C Manirakiza

**Affiliations:** 1 UT Southwestern Medical School, 5323 Harry Hines Boulevard, Dallas, TX, 75390-9003, USA; 2 Rwanda Cancer Relief, P.O BOX 4016, Kigali, Rwanda; 3 Dalla Lana School of Public Health, University of Toronto, 155 College St., Toronto, ON, Canada; 4 Wayne State Center for Urban Studies, 5700 Cass Ave, Detroit, MI, USA; 5 Rwamagana Provincial Hospital, P.O.Box 06, Rwamagana, Eastern Province, Rwanda; 6 School of Medicine, University of Global Health Equity, P.O.Box 6955, Kigali, Rwanda; 7 SingHealth Duke NUS, Singapore 169857, Singapore; 8 University of Medicine and Pharmacy C Davila, Dionisie Lupu Street, no. 37, Sector 2, Bucharest, 4192910, Romania; 9 African Medical Research and Innovation Institute, Tafelberg Rd 73, Cape Town 7550 SA; 10 Butaro Cancer Center of Excellence, Burera District, PO Box 59, Musanze, Rwanda; 11 Oncology Unit, Department of Medicine, King Faisal Hospital, KG 546 St, PO Box 2534, Kigali, Rwanda; 12 College of Medicine and Health Sciences, University of Rwanda, PO Box. 3286, Kigali, Rwanda

**Keywords:** colorectal cancer, Rwanda, Sub-Saharan Africa

## Abstract

**Background:**

The incidence of colorectal cancer (CRC) in sub-Saharan Africa (SSA) is rising, due to improving cancer registration efforts on one hand and an increasing westernisation of diets and lifestyle on the other as well as increasing rates of comorbidities.

**Methods:**

We present data for the clinical characteristics, pathology, treatments received, and survival outcomes of patients diagnosed with CRC at King Faisal Hospital (KFH) between January 2019 and May 2023. KFH is an urban tertiary hospital in Rwanda that provides chemotherapy and surgery to cancer patients. The data were extracted from electronic medical records, imaging and histopathology reports from the patient’s time of diagnosis. We plotted Kaplan–Meier estimation of survival, defined as the time from presentation to death, within the study period (2019–2023).

**Results:**

Seventy-four patients diagnosed with CRC with complete information were identified in the KFH oncology records. The mean age at diagnosis was 54.6 years, with ages ranging between 22 and 81 years. At diagnosis, 24 (32.4%) patients were less than 50 years old and 29 (39.2%) were females. The rectum (36.5%) was the most common tumour location, and 58.1 tumours were left-sided. Most patients presented with Stage III (41.9%) or IV (35.1%) disease. Adenocarcinoma was the most common histological type (98.6%) including adenocarcinoma not otherwise specified (NOS) (86.5%), mucinous adenocarcinoma (10.8%), signet ring cell carcinoma (1.4%) and followed by squamous cell carcinoma (1.4%). In terms of treatment, 19 (25.7%) patients received only chemotherapy, 43 (58.1%) patients received neo-adjuvant or adjuvant chemotherapy, 9 (12.2%) of patients received both neo-adjuvant and adjuvant chemotherapy, 49 patients (66.2%) underwent surgery and 17 (23%) patients also received radiation. At the end of the follow up period, 63 (85.1%) patients remained in surveillance, 10 (13.5%) patients died, and 1 (1.3%) patient was lost to follow up. Mean overall survival was 45.5 (SD ± 2.0) months.

**Conclusion:**

CRC patients presented at an advanced stage and required complex treatment regimens at KFH. Further epidemiologic and molecular research is needed to characterise CRC incidence and presentation at a national level in Rwanda as increasing westernisation continues to change the face of CRC in urban areas of SSA.

## Introduction

Colorectal cancer (CRC) is the third most diagnosed cancer and the second leading cause of cancer deaths worldwide, accounting for 9.1% of cancer deaths each year [1]. The incidence and mortality of CRC are expected to increase over the coming decades globally, with the largest relative increases in low and middle-income countries [2]. While data on CRC epidemiology in Sub-Saharan Africa (SSA) are limited, recent studies have shown an increasing incidence of cases in this region, with patients presenting at a younger age and a more advanced stage than in high-income countries [3]. This increase may be related to improved screening, detection, management modalities and cancer reporting, or increasing prevalence of modifiable risk factors with Westernisation of lifestyles and diets [3–6]. Studies in Eastern African countries of Tanzania and Kenya have documented increasing rates of CRC incidence, corroborating similar findings in the rest of the SSA region [5, 6]. Mean relative survival rates in SSA are low compared to other regions [7]. A meta-analysis of CRC overall survival rates in the region found a 5-year survival rate of 28%, compared to 5-year survival rates of between 56% and 66% reported in high-income countries [8].

Prior studies in SSA show a range of CRC incidence in patients under 40 years of age between 19% and 38%, compared to a range of 1%–9% in Western countries like the USA [4, 9]. The disproportionate rates of early onset CRC may be related to the relatively younger population in SSA as well as improving healthcare access and hereditary factors. In high-incidence countries, it is estimated that around 5% percent of CRC cases are caused by high penetrant inherited mutations such as familial adenomatous polyposis and Lynch Syndrome hereditary non-polyposis colorectal cancer, while a further 35% may be related to other genetic susceptibility [10, 11]. However, most CRC cases are sporadic and linked to dietary and lifestyle factors [12]. In SSA, urbanisation has been linked to dietary modifications, including a decrease in consumption of plant-based foods and staple carbohydrates, and an increase in the intake of meat and energy-dense, high-fat foods [13]. A study in Uganda showed an association between CRC incidence and individuals who lived in urban areas [14]. A known risk factor for CRC in urban settings is a westernised diet with high consumption of processed meat [15]. A higher incidence of CRC in urban areas than in rural areas has also been found in studies from the USA and the Middle East [16, 17]. In SSA, an urban residence has also been found to be associated with a higher incidence of CRC [18]. Urbanisation is associated with obesity due to a sedentary lifestyle, noncommunicable diseases such as diabetes and behavioural risk factors, in particular smoking and alcohol consumption, which are associated with CRC [19].

Rwanda is a low-income country located in SSA with a population of about 13.2 million and a gross domestic product of 13.1 billion USD as of 2022 [20, 21]. The median age in Rwanda is 21 years. An estimated 27.9% of people live in urban settings and 72.1% in rural settings [20]. There is a paucity of cancer data in Rwanda, given that the Rwandan National Cancer Registry was started in 2018 [22]. The most frequent cancer at the King Faisal Hospital Rwanda (KFHR) is breast cancer. It is not reflective of the overall figures in the Rwanda national cancer registry where cervical cancer is the most common, given that radiation therapy, essential in the treatment of locally advanced cervical cancer is not available at the hospital. The Globocan estimates that there were 8,835 new cancer cases in 2020 in Rwanda, with 173 new cases of colon cancer and 194 cases of rectal cancer [23]. Between 2010 and 2019, the total estimated CRC incidence in Rwanda increased an estimated 72% [3]. CRC caused an estimated 265 deaths in Rwanda in 2020, accounting for 4.4% of total cancer deaths in Rwanda that year [23]. Previous studies have shown that CRC patients presenting in Rwanda tend to be younger than in other regions outside of SSA, are more likely to be females, and are diagnosed with advanced disease [24–26]. 

Rwanda has made significant investments in improving cancer care in the country, with the establishment of the Butaro Cancer Center of Excellence (BCCOE) in 2012 and initiation of the National Cancer Control Plan (2020–2024) [22, 27]. More than 80% of Rwandans have access to affordable healthcare through the community-based health insurance system, Mutuelle de Sante [28]. However, many cancer drugs, especially newer, targeted drugs, are not affordable to the government and are not covered by community-based insurance [22, 29]. Currently, there are no screening guidelines for CRC in Rwanda, and as of 2019, there were only public hospitals in Kigali doing colonoscopies and sigmoidoscopy [22]. The Rwanda National Cancer Control Plan specified improving early detection of CRC with the development of national screening guidelines, training for healthcare workers, increased access to diagnostic technology, and improved public awareness of CRC signs and symptoms [27].

Rwanda-specific data on CRC epidemiology and treatment is essential for initiatives to understand the disease mechanisms, improve the diagnosis and management of CRC in Rwanda and to contribute on how lifestyle changes and westernisation of diets is changing the face of CRC in an urban setting in SSA. In this study we present data on CRC presentation, histopathology, treatment, and survival outcomes of patients who received CRC treatment at King Faisal Hospital (KFH), located in the capital city of Rwanda, Kigali, between 2019 and 2023.

## Methods

### Study design

This was a retrospective descriptive observational study including adult patients (>18 years old at diagnosis) who received treatment for CRC at KFH in Kigali, Rwanda between January 2019 and May 2023. The time period of interest of the study was chosen based on the likelihood of obtaining complete medical information from the records. Patients were identified from hospital medical records. Patient age, gender, stage at diagnosis, histopathology, treatment and survival details were extracted from patient charts, imaging and histopathology reports from the time of diagnosis and recorded in a single datasheet by the study team.

The KFHR is an academic private tertiary hospital in Kigali, Rwanda. The Oncology unit at the KFHR is one of the three clinics providing cancer care in Rwanda, the others being namely, the BCCOE and the Rwanda Cancer Center at the Rwanda Military Hospital. Cancer diagnostic capacity (Radiology and Pathology) exists at the three mentioned hospitals, with the addition of the Kigali and Butare University Teaching Hospitals, which provide oncology surgical care.

The KFHR has a colonoscopy facility and provides further imaging with computed tomography scan and MRI for staging purposes, not positron emitted tomography-computed tomography scan is available as of 2024. *Anatomic* Pathology services are available at the hospital. The department provides histology and immuno-histochemistry. Microsatellite status by Immuno-histochemistry started in 2024, and patients in this study did not benefit from this facility at the hospital. Molecular testing (for BRAF, KRAS, NRAS, p53 and mismatch repair (MMR) testing with PCR) is not available. These tests are requested and performed abroad, and patients are tested based on staging (stage IV) and failure of initial, primary treatment to allow for additional treatment options. Pathology services capacity is also present at the Rwanda Military Hospital, Kigali University Teaching Hospital (Kigali) and two other private centres. Nationally, The BCCOE and Butare University Teaching Hospital provide pathology services.

As of 2024, Radiation therapy is available only at the Rwanda Cancer Center of the Rwanda Military Hospital.

Four Rwandan oncologists were employed at the Oncology unit of the KFH at various times during the study period, providing oncology care to all cancer patients at KFH, including CRC patients. The individuals who receive cancer care at KFH must have private, government, or military insurance or pay out of pocket and therefore may be of a higher socioeconomic background than the general Rwandan population.

### Statistical analysis

Patients’ characteristics are described as mean with standard deviation for quantitative variables and as number and frequency (percentage) for categorical variables. Data were exported to SAS for analysis. Comparisons were determined using chi-square and student *T* test procedures. We measured survival as the time from the month of diagnosis to the month of death, regardless of cause, or loss to follow up, or censoring on 30th September 2023. We plotted survival curves using the Kaplan–Meier method. The log-rank test was used to assess the statistical differences in the observed survival curves by treatment intent. For all analyses, a *p*-value of  <  0.05 was considered statistically significant.

### Ethical considerations

This study was approved by the KFH Institutional Review Board and the University of Texas Southwestern Institutional Review Board.

## Results

There were 79 patients identified with confirmed diagnoses of CRC who received treatment at KFH between January 2019 and May 2023. Their data were retrieved from the electronic medical records. Of the 79 CRC patients identified, 5 were excluded because their treatment fell out of the study period or significant information was missing from their records. 74 patients were included in the analysis. These included 45 (60.8%) males and 29 (39.2%) females. The age at the time of diagnosis ranged between 22 and 81. The mean age for all sexes at diagnosis was 54.6 (SD ± 13.0 years). The mean age at diagnosis for women was 51.4 (SD ± 13.4 years) and the mean age for men was 56.6 (SD ± 12.5 years). Most patients (63.5%, *n* = 47) were diagnosed at KFH. See Supplement Table A for patient, tumour, and histopathology characteristics in table format.

Presenting signs and symptoms were recorded for 47 patients. Many patients were presented with multiple symptoms. The most frequently reported presenting symptoms included abdominal pain (61.7%), rectal bleeding (42.6%), unintended weight loss (34.0%) and constipation (31.9%).

**Figure 1.**
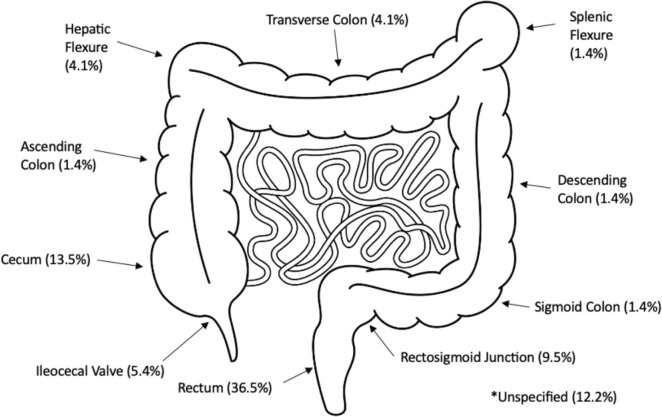
Anatomical tumour sites. Image from: www.istockphoto.com Purchased 01/03/2024

Figure 1 summarises the overall proportions of disease found per anatomical site across the colon. Over half (58.1%) of the tumours were left-sided, 19 (25.7%) were right-sided, 3 (4.1%) were transverse and 9 (12.1%) were in unspecified locations.

Disease staging was determined using the American Joint Committee on Cancer Tumor Node Metastasis (TNM) system. Two patients (2.7%) presented with Stage I disease, 13 (17.6%) with Stage II, 31 (41.9%) with Stage III, 26 (35.1%) with Stage IV disease, and staging was not recorded for 2 (2.7%) patients. The most common locations of metastases at the time of diagnosis were the liver and the lungs. The baseline serum carcino-embryonic antigen (CEA) was available for 44 patients with an average of 256.8 ng/mL.

Adenocarcinoma was the most common histological type (98.6%) including adenocarcinoma NOS (86.5%), mucinous adenocarcinoma (10.8%), signet ring cell carcinoma (1.4%), and squamous cell carcinoma (1.4%). The tumour histologic grade was I in 8.1% of patients, grade II in 45.9% of patients, grade III in 4.1% of patients, and not specified in 41.9% of cases.

Treatment was initiated with palliative intent for 26 (35.1%) patients, with curative intent for 46 patients (62.2%), and intent was not recorded for 2 patients (2.7%). A number of patients (13.5%) received some portion of their treatment abroad. For the 43 patients treated with curative intent with complete data, 39 (90.7%) received surgery and neo-adjuvant and/or adjuvant chemotherapy, 2 (4.7%) underwent upfront surgery and 2 (4.7%) received chemotherapy only (1 patient opted not to get surgery, and the other underwent surgery at an outside institution). Of the patients treated with curative intent, 12 (27.9%) also received radiotherapy. Out of the 26 patients treated with palliative intent, 10 (38.5%) received surgery and neo-adjuvant and/or adjuvant chemotherapy and 16 (61.5%) received chemotherapy only. Of these patients, 3 (11.5%) also received radiotherapy.

Of the 49 patients who underwent surgery, 2 (4.1%) had total colectomies, 15 (30.6%) had a right hemicolectomy, 7 (14.3%) had a left hemicolectomy, 1 (2.0%) had a rectosigmoidectomy, 4 (8.2%), had a sigmoidectomy, 4 (8.2%) had a lower anterior resection, 3 (6.1%) had an abdominoperineal resection, 1 (2.0%) had a small bowel resection and the specific procedure was not specified for 12 (24.5%) of patients

For the 67 patients who completed their first-line chemotherapy regimen, the number of cycles received in the first regimen was recorded for 52 patients. The average number of cycles received in the first chemotherapy regimen was 7.6 (± 3.2) cycles. Of the patients who received chemotherapy, 27 (40.3%) completed additional lines of chemotherapy. The average number of regimens completed by each patient was 1.5 (±1.1). The most common reason for beginning a second regimen of chemotherapy (for 11 patients) was due to disease progression, or persistent disease after their first regimen, while three patients changed regimens due to drug side effects, two for financial reasons, and one for compliance-related issues. Additionally, ten patients (37%) started a new chemotherapy regimen after disease recurrence. Treatment details are shown in Table 1.

The most frequently used first-line chemotherapies included the modified FOLFOX-6 regimen (5-Fluorouracil (5-FU), Leucovorin and Oxaliplatin) and CAPOX (Capecitabine and Oxaliplatin). The most frequently used second-line chemotherapies included XELIRI/CAPIRI (Capecitabine and Irinotecan), Capecitabine, FOLFIRI (5-FU, Leucovorin, Irinotecan), CAPOX and Irinotecan, often with the addition of Bevacizumab or Cetuximab.

Molecular testing was usually ordered for patients with upfront metastatic disease and patients presenting with progression on the first line of treatment, independently of the primary treatment intent.

**Figure 2.**
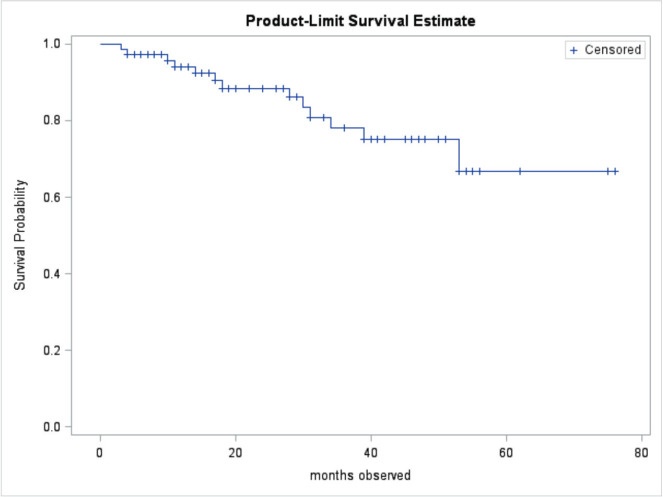
Overall survival curve.

**Figure 3.**
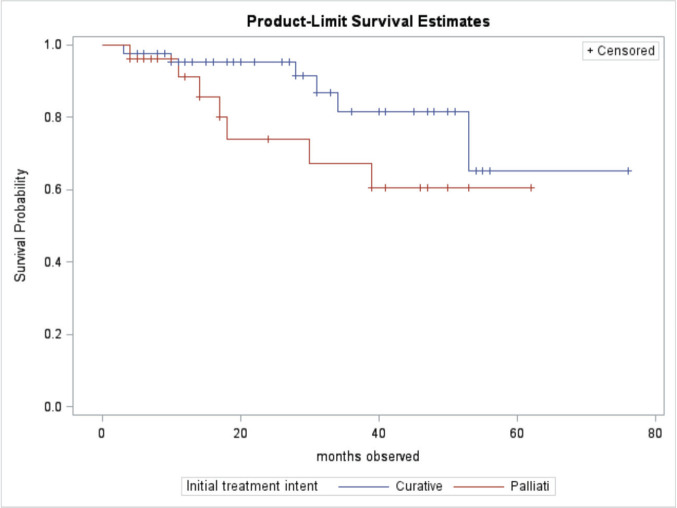
Comparison of survival curves by treatment intent.

At the end of the study period, 63 (85.1%) of patients were alive, 10 (13.5%) had died, and 1 (1.3%) was lost to follow up. Patient outcomes are shown in Table 2. Of the patients treated with curative intent, 84.8% were alive at the end of the study period while 73.1% of patients treated with palliative intent were alive at the end of the study period. There was no significant difference in mean survival outcome by treatment intent (*p* = 0.147). Mean overall survival, independent of treatment intent, was 45.5 ± 2.0 months. Mean survival for patients treated with curative intent was 47.9 ± 2.3 months. Mean survival for patients treated with palliative intent was 31.7 SD ± 2.8 months. Table 3 shows the survival outcome of patients based on stage at presentation. The correlation between stage at presentation and survival outcome was not statistically significant (*p* = 0.172). Survival curves are plotted in Figures 2 and 3.

## Discussion

CRC is a major public health problem. Globally, CRC is on the rise with increasing numbers of new cases occurring in low-middle income countries (LMICs), primarily in urban areas [3, 30]. This study aimed to characterise the demographics, histopathology, clinical presentation, and outcomes of CRC patients and to describe the CRC treatment patterns at KFH, while considering the patient population served by KFH.

Among 74 patients, we found that the mean age at diagnosis was 54.6 years with a wide age range between 22 and 81. This finding is similar to previous findings at the BCCOE and Kigali University Teaching Hospital (KUTH) with reported mean age at presentation of 52.5 and 54.3 years, respectively [24, 25]. The mean age of males at diagnosis was 56.6 years and the mean age of females at diagnosis was 51.4 years. The proportion of CRC patients under the age of 40 diagnosed at KFH was 13.4%, 18.9% of patients diagnosed were between the age of 40–49, while the remaining 67.6% were over the age of 50. Other studies conducted in nearby SSA countries have reported that 19%–38% of patients diagnosed with CRC are under the age of 40 [4]. The finding of a smaller proportion of CRC cases in patients under 40 years old at KFH (13.5%) as compared to previous studies in SSA corresponds with the population served by KFH. KFH is a private hospital and therefore tends to serve a patient population of a higher socioeconomic status, in contrast to some other hospitals providing cancer care in Rwanda such as BCCOE, where 58.1% of CRC patients lived in rural areas [25]. KFH patients primarily live in Kigali, an urban setting, and may have more access to lifestyles and diets associated with westernisation and therefore more exposure to risk factors that lead to higher incidences of CRC in countries like the USA. However, cancer awareness remains limited.

The role of genetic predisposition to developing CRC amongst our patients is not quite known. Genetic factors can impact the development of CRC in both familial and sporadic cases. Genetic alterations in susceptibility genes of varying penetrance have been linked to familial CRC. About one-third of patients with CRC in our cohort were under 50 years old. The National Comprehensive Cancer Network (NCCN) guidelines recommend a multigene panel test for all individuals with a diagnosis of CRC below the age of 50 years, regardless of somatic MMR gene status [31]. This is not commonly done in our practice, and only somatic characterisation is requested for patients who progress on the first line of treatment (FOLFOX and/or FOLFIRI). Approximately 12%–15% of CRCs exhibit deficient DNA mismatch repair (dMMR)/microsatellite instability (MSI), influencing prognosis and response to 5-FU-based adjuvant chemotherapy [32]. Identifying dMMR/MSI status, recommended for all resected CRCs, informs tailored treatment decisions, highlighting the importance of testing to guide precise therapeutic interventions.

A cross-sectional study in Zimbabwe of 101 CRC cases recruited in a tertiary hospital found that up to 3.3%–5.6% of patients were determined to have hereditary CRCs based on family history, germline, and somatic testing, corresponding to estimates from high incidence countries [9, 10, 33]. The prevalence of Lynch syndrome in the Zimbabwean study was 2%–4% which is also similar to rates in other high incidence countries [9, 34]. These findings underscore the importance of integrating genetic testing into our healthcare settings for early-onset CRC cases. By identifying individuals with hereditary predispositions, healthcare practitioners can implement targeted surveillance and preventive measures. This synthesis highlights the evolving landscape of CRC research in SSA and the imperative of incorporating genetic insights into clinical practice for improved patient outcomes.

Our finding that a majority of CRC patients at KFH were male contrasts with previous authors in Rwanda, who found a higher CRC incidence in female patients [24–26]. However, ‘it is in keeping’ with or ‘it supports’ previous findings in SSA of higher incidence of CRC in males, highlighting a difference between the patient populations served by different hospitals within Rwanda providing cancer care [3, 4]. We found a higher incidence of left-sided (58.1%) tumours with the rectum being the most common tumour location. These numbers, along with the lower rates of left sided-hemicolectomy surgeries highlight the growing surgical expertise in our settings and presentation at earlier stages of disease, as rectal cancer is approached surgically through localised, less extensive approaches either with or without neo-adjuvant treatment.

These numbers however do not explain the higher rates of right-sided hemi-colectomy seen in our cohort, which is based on the increased number of rectal cancer diagnoses, that are surgically approached through localised, less extensive approaches either with or without neo-adjuvant treatment. Our findings correspond to similar results found in Rwanda, of higher rates of rectal tumours at both BCCOE (74.2%) and CHUK (83.3%) [24, 25]. Our finding that left-sided tumours were more common in CRC patients at KFH is consistent with other literature from the region [4, 24]. Tumours on the left side are more frequently diagnosed in SSA. This could be because they are easier to investigate and diagnose, possibly due to being more accessible. Additionally, rectal tumours on the left side may show more symptoms, prompting patients to seek treatment earlier [4, 26]. Therapy responses vary greatly by tumour entity and location. Left-sided colorectal cancer is associated with a better prognosis and these patients may benefit from 5-FU-based regimens and targeted therapies, while right-sided colorectal cancer patients do not respond well to conventional chemotherapies but demonstrate more promising results with immunotherapies due to the high antigenic load of these tumours [35].

For all patients who survived, the mean survival period was 45.5 months (3.8 years). Mean survival time and its standard error were underestimated because the largest observation was censored, and the estimation was restricted to the largest event time. For patients treated with curative intent, the mean overall survival was 47.9 months (4.0 years), while the mean disease-free survival (defined as time from presentation to cancer recurrence, progression or death) in the study conducted at BCCOE was 22.2 months [25]. Our study looked at the overall survival of CRC patients; however, the contrast between KFH and BCCOE might be influenced by varying healthcare access and resources. At CHUK, another hospital in Kigali, the overall mean survival for CRC patients was 46 months [24]. Similar studies done in Tanzania, Ghana and Nigeria found median survival times of 9.4, 15 and 24.1 months, respectively [36–38]. Divergent median overall survival times in cancer studies across Rwanda, Tanzania, Ghana, and Nigeria indicate substantial regional disparities in CRC outcomes, likely influenced by distinct healthcare infrastructures and patient demographics. For patients treated with palliative intent, the mean survival was 31.7 months (2.6 years). However, this difference was not statistically significant. The TNM stage is crucial for determining CRC survival, yet stage-independent variability highlights the need to use robust biomarkers.

In our study, an analysis of patient presentations revealed that a substantial majority (76.1%), exhibited either stage III (41.9%) or stage IV (35.9%) CRC. This observation aligns with findings from analogous investigations conducted in other SSA countries. In studies conducted in Ghana, Tanzania and Uganda, the prevalence of late-stage CRC at presentation was 57%, 90% and 72.8%, respectively [38–40]. Our findings diverge from those reported in the United States, where disease awareness and advanced screening colonoscopy have led to a marked increase in the identification and removal of precancerous lesions, contributing to the diagnosis of CRC at earlier stages [41, 42]. This discrepancy underscores disparities in healthcare infrastructure and screening practices. Notably, the absence of widespread access to colonoscopy as a screening tool in Rwanda is a critical factor contributing to the presentation of patients with advanced-stage CRC. Unlike the United States, where screening measures have become integral to healthcare strategies, the limited availability of colonoscopy as a screening modality in Rwanda underscores the urgent need for enhanced diagnostic infrastructure and preventative healthcare initiatives in the region.

While data on symptom duration prior to presentation was rarely recorded in the patient records, many patients included in the study presented with severe symptoms and signs of abdominal obstruction requiring surgical resection. CRC patients at KFH received long-duration treatment regimens including multiple different chemotherapy drugs, surgery, and radiation highlighting the complexity and multi-disciplinary nature of CRC treatment. Many chemotherapy drugs are expensive and not widely available through the public health insurance program in Rwanda, which insures more than 80% of the total population.

## Limitations

Gaps in data collection were an important limitation of this study. There is no standard format for patient charts at KFH, therefore some data were not consistently available for all participants. Additionally, as KFH is a referral hospital, some patients were diagnosed elsewhere, making some of the details from their initial diagnosis unavailable at the time of treatment. Patients with significant information missing were excluded from the study. Another important limitation is that KFH does molecular testing only for patients presenting at stage IV and patients who progress on treatment. Our survival analysis did not adjust for competing comorbidities to deduct cancer-specific survival or mortality. Finally, KFH is a private hospital, in contrast to other hospitals providing cancer care in Rwanda, and therefore tends to serve a patient population of a higher socioeconomic status. This may have impacted the disease presentation, the treatments available and/or accessible to patients, and the survival outcomes of the patients. The results of this study may not be generalisable to the Rwandan population but are reflective of colon cancer disease patterns in a new, rising middle class in SSA at risk with changing lifestyles and diet.

## Conclusion

CRC patients at KFH frequently present at advanced stages, with a notable rise in individuals over 40, aligning with the increasing adoption of Western lifestyles and diets in urban areas like Kigali. To comprehensively address the CRC burden in Rwanda, it is imperative to conduct further epidemiological and molecular research at a national level and systematic screening. This research can provide initial insights into poorly characterised molecular changes and genomic alterations associated with CRC in SSA, facilitating the adoption of evidence-based best practices and policies. These efforts are essential not only for improving screening and early detection but also for advancing precision medicine and addressing the growing impact of Westernisation on CRC incidence and outcomes in urban areas of Rwanda. The integration of genetic testing and molecular research becomes critical in enhancing our understanding of CRC, contributing to the development of targeted interventions and personalised healthcare strategies to effectively combat the rising burden of CRC in the region.

## Recommendations

From this research, we recommend future research in molecular and genetic CRC testing, registry, improving population awareness of CRC, and increasing the availability of colonoscopies and screening in Rwanda.

## Funding

Margaret Baldwin was supported by the UT Southwestern Medical School Office Global Health.

## Conflicts of interest

All authors declare no conflicts of interest.

## Figures and Tables

**Table 1.** Details on types of treatment received (intent and modality).

Treatment characteristics	Number	Percent
Initial treatment intent (*n* = 74)		
Palliative (Stage IV disease)	26	35.1
Curative (Stage I–III disease)	46	62.2
Not specified	2	2.7
Travelled abroad for part of treatment (*n* = 74)		
Yes	10	13.5
No	64	86.5
Treatments received for patients with complete treatment data		
Curative intent: stage I–III disease (*N* = 43)		
Surgery only	2	4.7
Chemotherapy only	2	4.7
Neo-adjuvant + adjuvant chemotherapy + surgery	4	9.3
Neo-adjuvant chemotherapy + surgery	9	20.9
Adjuvant chemotherapy + surgery	26	60.5
Also received radiation	12	27.9
Palliative intent: stage IV (*N* = 26)		
Surgery only	0	0
Chemotherapy only	16	61.5
Neo-adjuvant + adjuvant chemotherapy + surgery	5	19.2
Neo-adjuvant chemotherapy + surgery	1	3.8
Adjuvant chemotherapy + surgery	4	15.4
Also received radiation	3	11.5
Types of surgery (*n* = 49)		
Total colectomy	2	4.1
Left hemicolectomy	7	14.3
Right hemicolectomy	15	30.6
Rectosigmoidectomy	1	2.0
Sigmoidectomy	4	8.2
Lower anterior resection (LAR)	4	8.2
Abdominoperineal resection (APR)	3	6.1
Small bowel resection	1	2.0
Unspecified procedure	12	24.5
Multiple chemotherapy regimen (*n* = 67)		
Yes	27	40.3
No	39	58.2
Unknown	1	1.5
Reasons for multiple chemotherapy regimens (*n* = 27)		
Disease progression/Persistent disease	11	40.7%
Drug side effects	3	11.1%
Financial	2	7.4%
Compliance	1	3.7%
Disease recurrence	10	37.0%

**Table 2.** Patient outcomes by treatment intent.

Patient outcomes (*n* = 74)	Number	Percent
Alive	63	85.1%
Died	10	13.5%
Lost to follow up	1	1.3%
Outcome by treatment intent		
Curative intent (*n* = 46)		
Alive	39	84.8%
Died	6	13.0%
Lost to follow up	1	2.2%
Palliative intent: (*n* = 26)		
Alive	19	73.1%
Deceased	7	26.9%

**Table 3.** Survival outcome by stage at presentation.

	Alive		Died		Total	
Stage	Number	Frequency	Number	Frequency	Number	Frequency
I	1	50	1	50	2	2.82
II	10	83.33	2	16.67	12	16.90
III	28	90.32	3	9.68	31	43.66
IV	19	73.08	7	26.92	26	36.62
Frequency missing = 3					71	100

*p* = 0.172

**Supplement Table A.** 

Characteristic (*N* = 74)	Number	Frequency (%)
Age at diagnosis, years		
<40	10	13.5
40–49	14	18.9
50–59	20	27.0
60–69	24	32.4
70–79	5	6.8
80	1	1.4
Sex		
Male	45	60.8
Female	29	39.2
**Signs and symptoms (*N* = 47)**	**Number**	**Frequency (%)**
Abdominal pain	29	61.7
Rectal bleeding	20	42.6
Weight loss	16	34.0
Constipation	15	31.9
Vomiting/Nausea	5	10.6
Diarrhoea	4	8.5
Anorexia	4	8.5
Back Pain	4	8.5
Fatigue	3	6.4
Melena	2	4.3
Anaemia	2	4.3
Many patients were presented with multiple symptoms, thus total % >100.		
**Histopathology characteristics (*N* = 74)**	**Number**	**Frequency (%)**
Type		
Adenocarcinoma	73	98.6
NOS	64	86.5
Mucinous	8	10.8
Signet ring cell	1	1.4
Squamous cell carcinoma	1	1.4
Grade		
I	6	8.1
II	34	45.9
III	3	4.1
Unknown	31	41.9
**Tumour locations (*N* = 74) **	**Number**	**Frequency (%)**
Mass location		
Left	43	58.1
Right	19	25.7
Transverse	3	4.1
Unspecified	9	12.2
Anatomic site		
Rectum	27	36.5
Rectosigmoid junction	7	9.5
Sigmoid colon	7	9.5
Descending colon	1	1.4
Splenic flexure	1	1.4
Transverse colon	3	4.1
Hepatic flexure	3	4.1
Ascending colon	1	1.4
Cecum	10	13.5
Ileocecal valve	5	5.4
Colon (not specified)	9	12.2

## References

[1] Sung H, Ferlay J, Siegel RL (2021). Global cancer statistics 2020: GLOBOCAN estimates of incidence and mortality worldwide for 36 cancers in 185 countries. CA: Cancer J Clin.

[2] Morgan E, Arnold M, Gini A (2022). Global burden of colorectal cancer in 2020 and 2040: incidence and mortality estimates from GLOBOCAN. Gut.

[3] Katsidzira L, Gangaidzo I, Thomson S (2017). The shifting epidemiology of colorectal cancer in sub-Saharan Africa. Lancet Gastroenterol Hepatol.

[4] Awedew AF, Asefa Z, Belay WB (2022). Burden and trend of colorectal cancer in 54 countries of Africa 2010–2019: a systematic examination for Global Burden of Disease. BMC Gastroenterol.

[5] Herman A, Hawkins AT, Misso K (2020). Colorectal cancer in Northern Tanzania: increasing trends and late presentation present major challenges. JCO Glob Oncol.

[6] Parker RK, Ranketi SS, McNelly C (2019). Colorectal cancer is increasing in rural Kenya: challenges and perspectives. Gastrointest Endosc.

[7] Gullickson C, Goodman M, Joko‐Fru YW (2021). Colorectal cancer survival in sub‐Saharan Africa by age, stage at diagnosis and human development index: a population‐based registry study. Int J Cancer.

[8] Hassen HY, Hussien FM, Hassen AM (2022). Survival pattern of colorectal cancer in Sub-Saharan Africa: a systematic review and meta-analysis. Cancer Epidemiol.

[9] Katsidzira L, Vorster A, Gangaidzo IT (2019). Investigation on the hereditary basis of colorectal cancers in an African population with frequent early onset cases. PLoS One.

[10] Jasperson KW, Tuohy TM, Neklason DW (2010). Hereditary and familial colon cancer. Gastroenterology.

[11] Graff RE, Möller S, Passarelli MN (2017). Familial risk and heritability of colorectal cancer in the nordic twin study of cancer. Clin Gastroenterol Hepatol.

[12] Murphy N, Moreno V, Hughes DJ (2019). Lifestyle and dietary environmental factors in colorectal cancer susceptibility. Mol Asp Med.

[13] Vorster HH, Kruger A, Margetts BM (2011). The nutrition transition in Africa: can it be steered into a more positive direction?. Nutrients.

[14] Wismayer R, Kiwanuka J, Wabinga H (2022). Risk factors for colorectal adenocarcinoma in an indigenous population in East Africa. Cancer Manag Res.

[15] Bouvard V, Loomis D, Guyton KZ (2015). Carcinogenicity of consumption of red and processed meat. Lancet Oncol.

[16] Coughlin SS, Richards TB, Thompson T (2006). Rural/nonrural differences in colorectal cancer incidence in the United States, 1998–2001. Cancer.

[17] Mostafa E, Yavari P, Vahedi H (2021). Urbanization levels and its association with prevalence of risk factors and colorectal cancer incidence. Iran J Public Health.

[18] Katsidzira L, Gangaidzo IT, Makunike-Mutasa R (2019a). A case-control study of risk factors for colorectal cancer in an African population. Eur J Cancer Prev.

[19] Singh GK, Miller BA, Hankey BF (2003). Area Socioeconomic Variations in U.S. Cancer Incidence, Mortality, Stage, Treatment, and Survival, 1975–1999. NCI Cancer Surveillance Monograph Series, Number 4 (Bethesda: National Cancer Institute).

[20] National Institute of Statistics of Rwanda (2023). The Fifth Rwanda Population and Housing Census, Thematic Report: Population Projections.

[21] International Monetary Fund (n.d.) Rwanda Datasets.

[22] Rubagumya F, Costas-Chavarri A, Manirakiza A (2020). State of cancer control in Rwanda: past, present, and future opportunities. JCO Glob Oncol.

[23] International Agency for Research on Cancer (2020). Rwanda: Fact Sheets.

[24] Uwamariya D, Ruhangaza R, Rugwizangoga B (2022). Pathological characteristics, prognostic determinants and the outcome of patients diagnosed with colorectal adenocarcinoma at the University Teaching Hospital of Kigali. Can J Gastroenterol Hepatol.

[25] Fadelu T, Sebahungu F, Diasti K (2020). Patient characteristics and outcomes of colorectal cancer (CRC) at Butaro Cancer Center of Excellence (BCCOE): results from a retrospective cohort. J Clin Oncol.

[26] Manirakiza F, Rutaganda E, Yamada H (2023). Clinicopathological characteristics and mutational landscape of APC, HOXB13, and KRAS among Rwandan patients with colorectal cancer. Curr Issues Mol Biol.

[27] Republic of Rwanda Ministry of Health (2020). Rwanda National Cancer Control Plan 2020-2023.

[28] World Health Organization (2019). Rwanda: the Beacon of Universal Health Coverage in Africa.

[29] Kizub DA, Naik S, Abogan AA (2022). Access to and affordability of World Health Organization essential medicines for cancer in Sub-Saharan Africa: examples from Kenya, Rwanda, and Uganda. Oncologist.

[30] Graham AM, Adeloye D, Grant L (2012). Estimating the incidence of colorectal cancer in Sub-Saharan Africa: a systematic analysis. J Glob Health.

[31] Alam S (2022). New NCCN Guidelines Recommend Genetic Testing for All CRC Patients.

[32] Jin Z, Sinicrope FA (2021). Prognostic and predictive values of mismatch repair deficiency in non-metastatic colorectal cancer. Cancers.

[33] Hampel H (2009). Genetic testing for hereditary colorectal cancer. Surg Oncol Clin North Am.

[34] Hampel H, Frankel WL, Martin E (2008). Feasibility of screening for Lynch syndrome among patients with colorectal cancer. J Clin Oncol.

[35] Baran B, Mert Ozupek N, Yerli Tetik N (2018). Difference between left-sided and right-sided colorectal cancer: a focused review of literature. Gastroenterol Res.

[36] Agyemang-Yeboah F, Yorke J, Obirikorang C (2018). Colorectal cancer survival rates in Ghana: a retrospective hospital-based study. PLoS One.

[37] Sharma A, Alatise OI, Adisa AO (2019). Treatment of colorectal cancer in Sub‐Saharan Africa: results from a prospective Nigerian hospital registry. J Surg Oncol.

[38] Lulabuka N, Dharsee N, Kahesa C (2019). Clinical-pathological characteristics of colorectal carcinoma and factors influence 2-years survival among patients attending ocean road cancer Institute Dar es Salaam Tanzania. Open J Gastroenterol.

[39] Yeboah FA, Yorke J, Obirikorang C (2017). Patterns and presentations of colorectal cancer at Komfo-Anokye teaching hospital Kumasi, Ghana. Pan Afr Med J.

[40] Wekha G, Ssewante N, Iradukunda A (2021). Colorectal cancer in Uganda: a 10-year, facility-based, retrospective study. Cancer Manag Res.

[41] Saluja S, Alatise OI, Adewale A (2014). A comparison of colorectal cancer in Nigerian and North American patients: is the cancer biology different?. Surgery.

[42] Wernli KJ, Hubbard RA, Johnson E (2014). Patterns of colorectal cancer screening uptake in newly eligible men and women. Cancer Epidemiol Biomarkers Prev.

